# A comparative analysis of the oral microbiome of Amish and non-Amish individuals to strengthen our understanding of variation within the oral microbiome

**DOI:** 10.1371/journal.pone.0350558

**Published:** 2026-06-24

**Authors:** Debra L. Wohl, Phillip T. Belder, Braxton D. Mitchell

**Affiliations:** 1 Department of Biological & Environmental Sciences, School of Sciences and Health, Elizabethtown College, Elizabethtown, Pennsylvania, United States of America; 2 Department of Medicine, University of Maryland School of Medicine, Baltimore, Maryland, United States of America; 3 Geriatrics Research and Education Clinical Center, Baltimore Veterans Administration Medical Center, Baltimore, Maryland, United States of America; University of Insubria, ITALY

## Abstract

More than 700 phylotypes associated with the oral cavity collectively comprise the oral microbiome. Study of microbiomes has advanced our understanding of human health. Little is known about the oral microbiome of the Old Order Amish population, a distinct ethnoreligious group who choose to stay separate from mainstream society to preserve their traditional, faith-based way of life. This research was to generate a novel characterization of the Amish oral bacterial microbiome and, using a comparative study design, provide metagenomic analyses of potential variations between generated profiles of the Amish and non-Amish. Next-generation sequencing of 16S rRNA genes of supragingival plaque and saliva samples was used. Analysis between oral health habits from surveys (e.g., fluoride use, frequency of dental visits) and markers within the microbiomes were used to assess the extent of variation due to oral health habits or other factors. Samples were analyzed from 14 Amish and 13 non-Amish individuals. Using non-parametric analyses, alpha and beta diversity were measured to assess core microbiomes, abundance, and sample dissimilarity. Compared to non-Amish, Amish experienced significantly lower frequency of dental visits (p < 0.001) and fluoride use (p < 0.001), but no difference in frequency of teeth brushing (p = 0.198) was observed. Alpha-diversity of observed species differed significantly between Amish and non-Amish samples (H = −3.89, p = 0.002). Beta-diversity which accounted for relative taxon abundance and presence, as well as other metadata such as fluoride use, frequency of dental visits, and teeth brushing indicated, for both saliva and plaque, samples clustered by grouping and their covariates. The five primary phyla typically associated with the oral microbiome were the dominant phyla in both Amish and non-Amish individuals, although Proteobacteria were proportionally fewer in Amish samples. We conclude the oral microbiome between the Old Order Amish and rural non-Amish are distinctly different, which may reflect observed differences in lifestyle and oral health habits.

## Introduction

Within the oral cavity is a community of commensal, symbiotic, and pathogenic microorganisms, which are collectively referred to as the oral microbiome. The oral cavity, which includes the teeth, gingival sulcus, attached gingiva, tongue, cheek, lip, and both the hard and soft palates, is a complex environment with great variation due to variables such as temperature, pH, salivary flow, salinity, and atmospheric gases [1]. Additionally, the oral cavity is a critical entry point to the human body. It is through this entry point that food and air gain access to the digestive tract and respiratory system, respectively. Microorganisms present within the oral cavity may be transient. Other microorganisms persist in highly selective microenvironments and have been associated with infectious disease such as tooth decay, periodontitis, endodontis, halitosis, and tonsillitis [1–4]. The oral microbiome has also been linked to outcomes such as preterm birth, heart disease, diabetes, and pneumonia [5,6].

Cultivation-independent methods, primarily using the 16S rRNA gene and the Human Microbiome Project (HMP,) led to a significant increase in our understanding of the oral microbiome and its role in human health. To date, more than 700 species or phylotypes associated with the oral microbiome have been curated and annotated in the extended Human Oral Microbiome Database (eHOMD) [7]. While the oral cavity maintains a diverse and abundant microbial community, the Phyla of bacteria that numerically contribute the most to the oral microbiome are the Actinobacteria, Bacteroidetes, Firmicutes, Fusobacteria, and Proteobacteria [2,3,8,9]. Many of these microorganisms play an important role in pathogen resistance by modifying the immediate environment or limiting attachment sights, while others contribute to acidification and influence the progression of disease. For example, *Streptococcus*, a genus found within the Firmicutes, includes both the well-documented contributor to the progression of dental caries *S. mutans* and *S. gordonii,* an antagonist of *S. mutans* [2,3,10,11].

Although there is disagreement on the temporal stability of the oral microbiome, healthy biofilms in the oral cavity regardless of dentition state have been shown to include *Corynebacterium*, a primary colonizer of the buccal surface, along with *Streptococcus, Capnocytophaga*, *Fusobacterium*, and *Actinomyces* [12–14]. *Neisseria* and *Prevotella* are both found to be abundant as well, although their association with the biofilm appears to be more loosely connected. Other genera associated with healthy oral microbiomes include *Veillonella*, *Porphyromonas*, *Treponema*, *Haemophilus*, *Preptostreptococcus*, and *Staphylococcus* [12], whereas *Streptococcus anginosus*, *Campylobacter*, *Tannerella*, and *Actinomyces actiomycetemimitas* are prevalent in individuals with disease progression in the oral cavity [6,12].

In addition to healthy and diseased states of the oral cavity, species richness and taxonomic distributions associated amongst individuals has been shown to vary by ethnicity, genetics, and lifestyle habits [14–16]. While research supports the existence of a core microbiome associated with the oral cavity, there are significant differences in the composition of the oral microbiome across hosts [2,9,15,17]. For example, Clemente et al. [17] found the oral microbiome of uncontacted Amerindians was dominated by similar phyla to those of developed Americans; however, they also found significantly greater abundances of *Prevotella*, *Fusobacteria*, and *Veillonella* in this Amerindian population. Another study found within the same Ecuadoran ethnic population that the gut microbiome differed in species richness and taxonomic distributions along an economic gradient [18].

The Old Order Amish population of Lancaster County, PA, USA is a founder population originating from northern Europe in the early 18^th^ century. The Lancaster settlement includes ~38,000 individuals. The Amish are a distinct ethnoreligious group who are very socially cohesive, choosing to stay separate from mainstream society to preserve their traditional, faith-based way of life. Their lifestyle is relatively homogeneous. A previous study demonstrated that the oral health knowledge and oral health habits of the Amish population is significantly lower than in the general population, with many Amish perceiving little need for dental care at all in 1988 [19]. Epidemiological studies performed by Bagramian et al. [19] demonstrated significantly higher incidence of oral disease in rural Amish compared to rural non-Amish adults. Furthermore, oral hygiene behavior scores were inversely correlated with gingivitis and periodontal disease [20]. This study by Bagramian et al. predates our current understanding of periodontal disease but demonstrates that unaccounted factors may influence the polymicrobial origin of Amish periodontal disease [20]. Although several molecular studies have been conducted on the gut microbiota in Old Order Amish, few to no molecular analyses have been conducted on the microbiota of the oral cavity, which may improve understanding of Amish periodontal disease [21].

The goal of this research is to generate a novel characterization of the Amish oral cavity bacterial microbiome and provide metagenomic analyses of potential variations between generated bacterial profiles of the Amish and non-Amish population. Based on the differences in diet, oral health habits, environmental exposures, and genetic composition, the hypothesis is that there will be a significant difference between the two microbiomes. We have performed next-generation sequencing of supragingival plaque and saliva samples to determine variation in taxonomical distribution. We further related oral health habits with pathogenic markers within the microbiomes.

## Materials, subjects, and methods

### Sample collection

We collected oral plaque and saliva samples from 21 Amish and 30 non-Amish individuals from Lancaster County, Pennsylvania, United States. No pre-visit preparation was required for the study. Amish volunteers aged 18 years or older from the community were recruited April 6, 2016, through April 8, 2016, by trained staff at the University of Maryland Amish Research Clinic [22]. Non-Amish subjects from the Lancaster community 18 years or older scheduled for routine dental care were recruited December 2, 2015, through December 8, 2015, by trained staff at White Family Dental. To test our hypothesis, we excluded from analyses individuals self-reporting a history of periodontal disease, oral cancer, dentures, or tobacco use in the last 5 years. Thereby, for this exploratory and comparative study, we analyzed data from 14 Amish and 13 non-Amish individuals.

In accordance with the Institutional Review Boards (IRB) of Elizabethtown College [FWA00020368] and the IRB of University of Maryland, Baltimore [FWA00007145], written informed consent was obtained by individuals at White Family Dental and waived at the Maryland Amish Research Clinic. For those willing to participate in the study, data collection consisted of an oral health survey first, and then the collection of biological specimens. The oral health questionnaire included questions about age, sex, and frequency of dental visits, teeth brushing, fluoride use, and use of mouthwash.

Plaque along the entire supragingival buccal surface of the teeth was lightly scraped via 5 mm disposable dermal curette (Acuderm Inc., Ft. Lauderdale, Florida, USA). Plaque was transferred immediately to a nylon FLOQSwab (COPAN Flock Technologies, Brescia, Italy). Swabs were torn at its perforation and placed within a 10 mL conical tube containing 5 mL of 0.85% saline (NaCl) solution [23]. Saliva samples were obtained via mouth rinse of 10 mL of 0.85% saline and subsequent expectoration into a 50 mL conical tube [24]. To ensure complete lack of DNA and DNase enzymes, a sterile saline solution was prepared using autoclaved dH_2_O, using the Milli-Q Integral 10 water purification system. Two negative controls were generated using only sterile saline and no biological sample collection. Samples were placed on ice immediately after collection until DNA extraction.

### DNA extraction and amplification

Plaque samples were vortexed for 30 s to release sample collection from swab into solution. Similarly, saliva samples were vortexed briefly to homogenize the sample prior to processing. To proceed, plaque samples were centrifuged using the Sorvall RC 5C Plus centrifuge at 4300 RPM for 3 min; saliva samples (i.e., 50 mL conical tube samples) were centrifuged at 2300 rpm for 3 min. Supernatant was removed until 1 mL remained. Tubes were vortexed to re-suspend for 15 s. DNA extraction was performed utilizing the MO BIO Bacteremia DNA Isolation Kit (Qiagen) with the substitution of 50 μL of dH_2_O as the final step. Adequate DNA concentrations of samples were confirmed using Qubit 3.0 Fluorometer and its designated protocol. Extracted DNA was then stored at −20°C for further analysis.

PCR amplification conditions were selected on existing protocols to amplify the V4 region of the 16S rRNA gene. All PCR reactions were performed in duplicate per each sample collected. One unique barcoded reverse primer was used for each duplicate PCR reaction. The PCR master mix contained *TaKaRa Ex Taq* polymerase (0.125 μL, final: 0.625U/rxn), 10X *Ex Taq* Buffer (2.5 μL, final concentration: 1X), dNTP mixture (2 μL, final concentration: 0.8mM), and 515F forward primer (1 μL). Each PCR reaction consisted of 1.0 μL of template DNA, 5.625 μL of aforementioned master mix, PCR grade H_2_O (17.375 μL), and 1.0 μL 806rcbc reverse primer [primer #s: 768–863]. Blanks used PCR grade H_2_O in lieu of template DNA.

Primers were obtained from Integrated DNA Technologies, Inc. (Coralville, IA, USA) and remaining PCR master mix components were obtained from TAKARA BIO, Inc. (Shiga, Japan). Two PCR negative controls were generated using PCR grade H_2_O in lieu of sample DNA. PCR Thermocycling conditions were 94C for 3 min, 35 cycles of 94C for 45 sec, 50C for 60 sec, and 72C for 90 sec, with a final extension of 72C for 10 min. Amplicons were stored at −20°C until sequencing.

### DNA sequencing and sequencing analysis

Sequencing libraries for each extracted sample were performed off-site at the HHMI Juniata College Core Facility (Selinsgrove, PA, USA) using the Illumina MiSeq Targeted Sequencing platform, yielding up to 300 base pair read lengths. Data from the Juniata Core Facility was analyzed using the open-source Quantitative Insights into Microbial Ecology (QIIME 1.9.1) statistical package [25]. The data from forward and reverse primer reads were merged to generate the full-length 254 bases read sequences. The sequences were quality filtered at AvgQ values (Phred scores) above 30 and AvgEE values below 0.5 error rate. The read lengths were subsequently truncated at 252 bases, the length of the V4 region.

Metadata containing survey data and sample codes were integrated into the sequences and validated to ensure no errors. Operational taxonomic units (OTUs) were clustered using open reference OTU picking using the UCLUST algorithm implements in the USEARCH protocols within QIIME. Chimera sequences were identified and removed from the sequence libraries to prevent diversity inflation. Taxonomy was assigned to the OTUs and filtered based on number of observations. Alpha diversity rarefactions were developed for each metadata variable using QIIME. Reads were subsampled without replacement to a depth of 10 000 sequences per sample. Samples with counts below this threshold were excluded from downstream diversity analyses. Alpha rarefaction curves were generated to plot number of observed OTUs against the number of sequences sampled. We used the nonparametric Kruskal-Wallis test to compare alpha diversity metrics (e.g., observed species, Heip’s evenness) to determine differences in samples grouped by population (i.e., Amish, non-Amish) and type (i.e., saliva, plaque).

Beta diversity was calculated after removal of singleton OTUs from the unfiltered OTU table. Normalization was performed via cumulative sum scaling (CSS) in order to correct bias within beta diversity computation. Principle Coordinates Analysis (PCoA) generated plots in which data point clustering could be observed. To quantitatively determine the metadata parameters (i.e., including variables such as fluoride use and dental visits) describing differences in community structure (e.g., Unifrac distances), we used the non-parametric multivariate statistical test, analysis of variance using distance matrices (ADONIS), to test for significant differences between two or more groups of samples.

Differences in OTU frequencies between samples grouped by population (i.e., Amish, non-Amish) and type (i.e., plaque, saliva) were analyzed using Kruskal-Wallis. Proportions of OTUs with Bonferroni corrected p-values ≤ 0.05 were considered significantly different between groups. Given the small sample sizes and to ensure true differences, the core microbiome was calculated using only OTUs present in non-zero concentration within all samples, 100%, of a particular group (e.g., Amish).

## Results

### Subject and survey characteristics

The median ages of the 14 Amish and 13 non-Amish study participants were 47.6 and 46.1 years, respectively (Table 1). There was no significant difference in sex distribution between the two populations, although both samples included predominantly women (Table 1). Amish reported fewer dental visits, or a longer interval since last visit, than non-Amish (p < 0.001). Fluoride, commonly used to guard against dental caries by reducing demineralization of enamel, was not present in either well or most public waters within our study area. Because individuals may choose fluoride supplementation by using commercially available toothpastes with fluoride, we included this question in our survey. Amish individuals reported less fluoride use than non-Amish individuals (p = 0.014; Table 1). The frequency of teeth brushing did not differ significantly between the two groups (p = 0.198).

**Table 1:** Study population characteristics gathered from the Oral Health Questionnaire.

Characteristics	Amish (n = 14)	Non-Amish (n = 13)	P-value
Age (years)	47.6	46.1	0.75
Sex (male)	11%	20%	0.35
**Dental Visits (n)**			**<0.001**
≤ 0.5 years	0	7	
> 0.5–2 years	3	6	
> 2–5 years	6	0	
≥ 5 years	5	0	
Teeth Brushing (n)			0.198
Daily	9	12	
1–6 times per week	5	1	
< 1 time per week	0	0	
**Fluoride (n)**			**0.014**
Daily	3	10	
1–6 times per week	1	0	
< 1 time per week	10	3	
Mouthwash (*no data for 1 non-Amish)			0.933
Daily	5	5	
1–6 times per week	3	2	
< 1 time per week	6	5	

Statistically significant relationships are bolded. Chi-Square test for independence was used.

### Sequencing and data analyses

A total of 4.7 million sequences with a read length of 252 bases were retained after sequences were paired, filtered, trimmed, and both chimeras and singletons were removed. The average sample sequence count was 89 854 ± 42 667 per sample.

**Fig 1:**
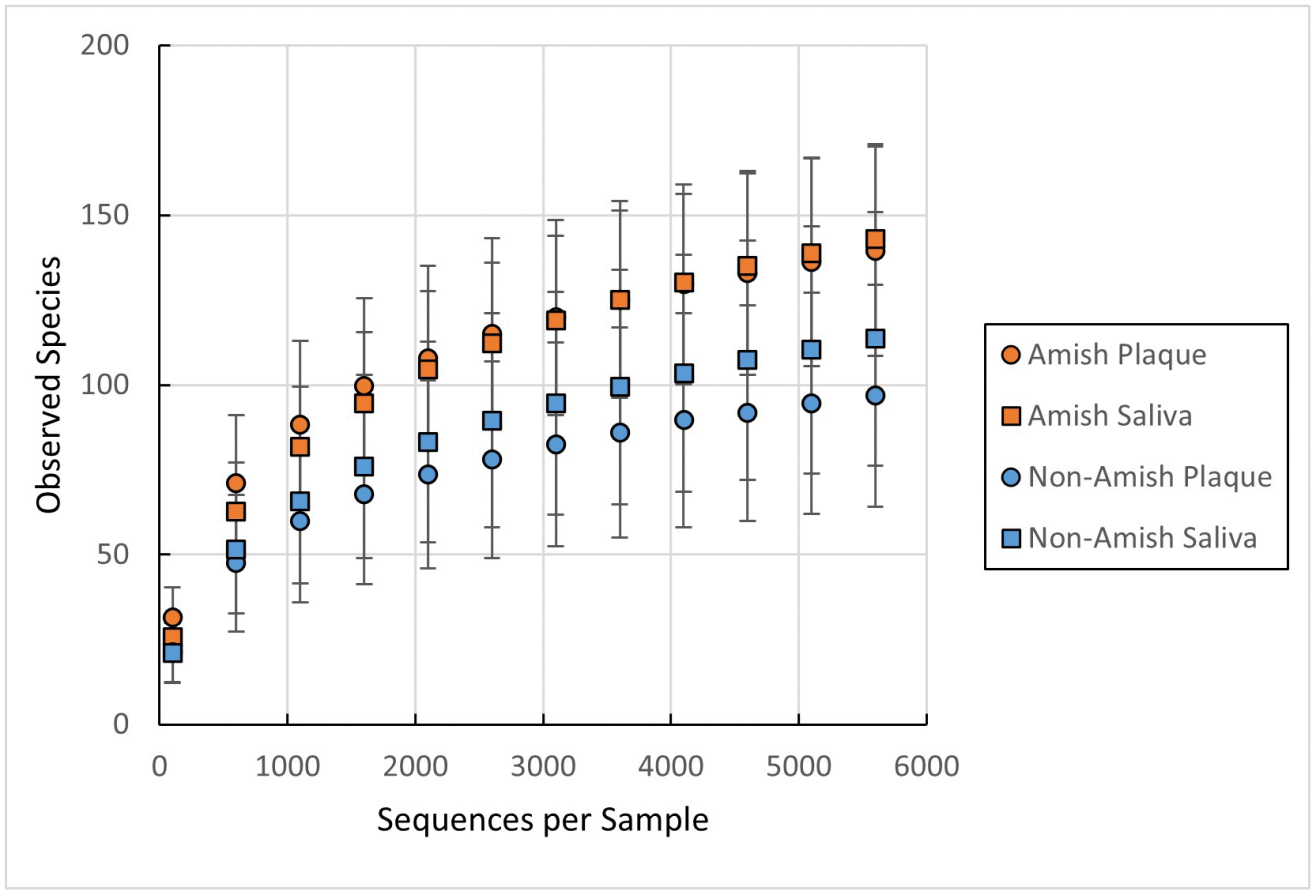
Rarefaction curves of baseline operational taxonomic unit (OTUs) prevalence rates according to number of reads. Comparisons include each group (Amish, non-Amish) by type sample (saliva, plaque). Differences between non-Amish plaque samples differed significantly from Amish saliva (H = −3.73, p = 0.006) and Amish plaque (H = −2.19, p = 0.024) samples. Orange symbols represent Amish samples; Blue symbols represent non-Amish samples; Circles designate plaque; Squares designate saliva.

Alpha-diversity, or within sample variation, of observed species differed significantly between groups, i.e., Amish and non-Amish samples (H = −3.89, p = 0.002). Non-Amish plaque samples had significantly different species richness than either Amish plaque or Amish saliva samples (H = −2.19, p = 0.024; H = −3.73, p = 0.006 respectively). No other significant differences between group (i.e., Amish, non-Amish) by type (i.e., plaque, saliva) interactions were found. Rarefaction, or subsampling, of the data supported adequate sampling depth for richness, phylogenetic distance (PD), and rare taxa. Observed species rarefaction curves show non-Amish samples have fewer OTUs than either the plaque or saliva samples of the Amish (Fig 1).

### Beta diversity and group significance

**Fig 2:**
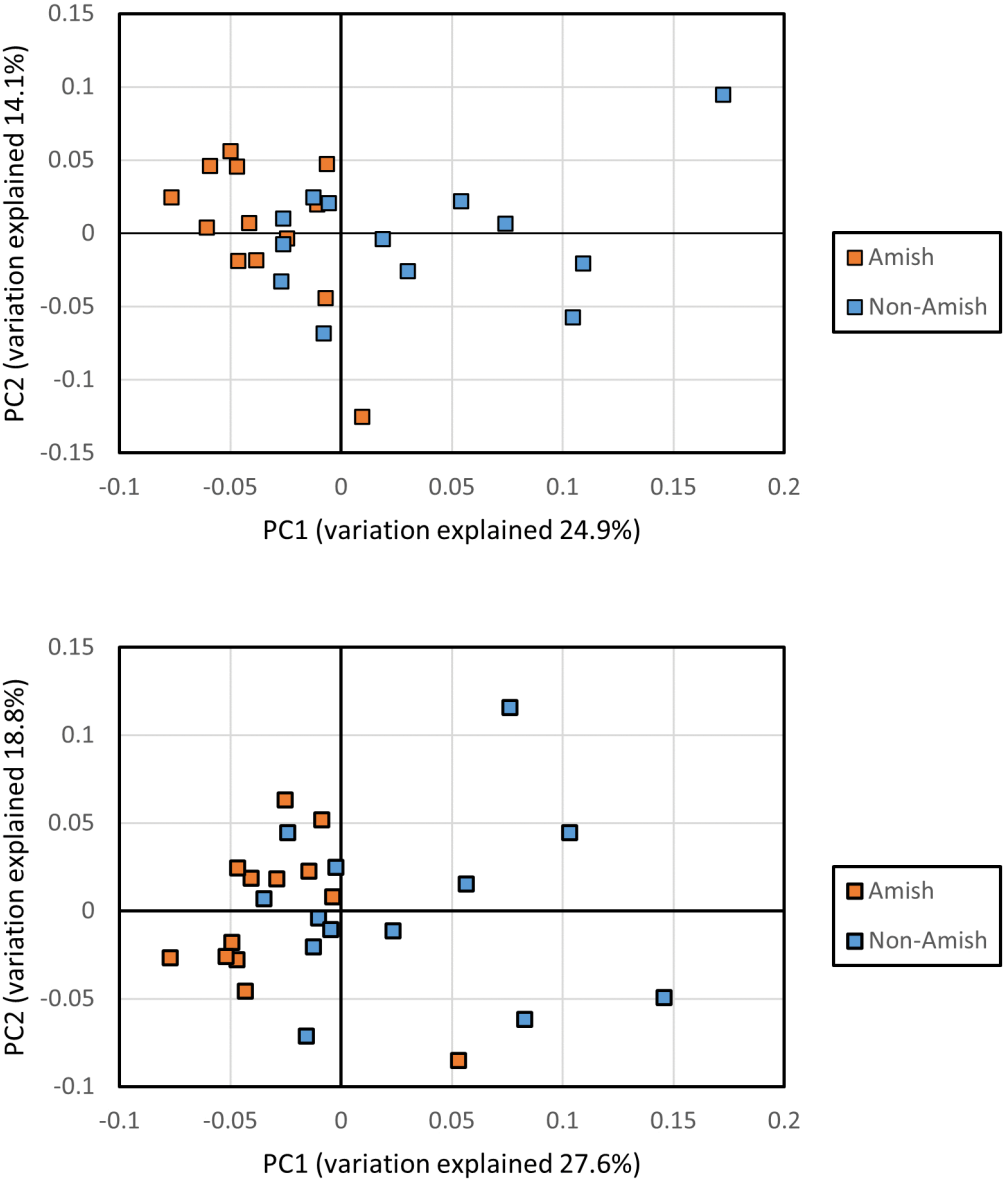
PCoA clustering analysis shown as 2D plots of (A) Plaque samples and (B) Saliva samples. Orange squares represent Amish samples; Blue squares represent non-Amish samples.

We used UniFrac weighted distances and PCoA to compare samples. Analyses included relative taxon abundance and presence, as well as metadata collected from surveys including age, sex, dental visit frequency, and fluoride and mouthwash use. The plaque samples displayed distinct clustering by group, Amish or non-Amish, as well as the covariates fluoride use and dental visits with 24.9% of the variation explained by the primary axis and 14.1% explained by the secondary axis (Fig 2a). Saliva samples also showed clustering by group and the covariates fluoride use and dental visits with 27.7% of the variation explained by the primary axis and 18.8% explained by the secondary (Fig 2b).

Analyses using ADONIS to compare phylogenetic composition between groups indicated a significant difference in taxonomic composition between Amish and non-Amish samples (F = 4.34, p < 0.001). Phylogenetic composition in plaque and saliva samples were also significantly different (F = 8.07, p < 0.001). The composition and distribution of OTUs between group by type interactions also differed significantly (F = 4.81, p = 0.001). However, there were no significant differences in phylogenetic composition between samples grouped by sex (F = 1.35, p = 0.154) or age (F = 1.24, R^2^ = 0.024, p = 0.214).

**Fig 3:**
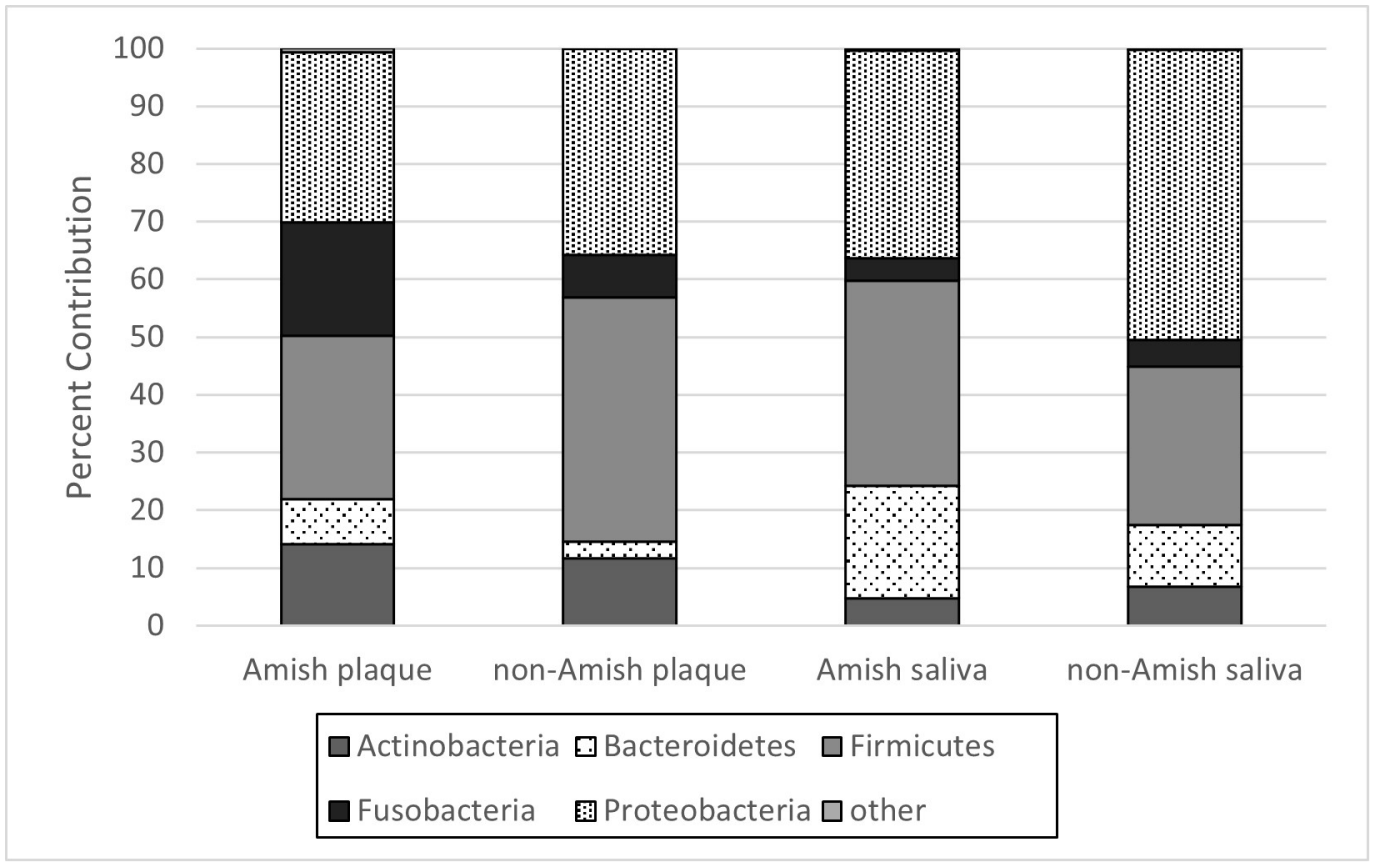
The relative contribution of five most abundant bacterial phyla contributing to Amish and non-Amish plaque and saliva samples. The phyla include Actinobacteria, Bacteroidetes, Firmicutes, Fusobacteria, and Proteobacteria Data are presented as stacked bars.

The five primary phyla typically associated with the oral microbiome, i.e., Actinobacteria, Bacteroidetes, Firmicutes, Fusobacteria, and Proteobacteria, were the dominant phyla in both Amish and non-Amish individuals. In Amish plaque samples, together the Bacteroidetes Actinobacteria, and Fusobacteria contributed 50.20% to the composition of the samples, whereas in the non-Amish plaque samples these same three phyla contributed 56.85% (Fig 3). Conversely, in the Amish saliva samples the three phyla contributed a greater percentage to the total composition than the non-Amish saliva samples (i.e., 59.71% and 44.89% respectively). The Proteobacteria, when compared between the Amish and non-Amish samples, were relatively more abundant in both the non-Amish plaque and saliva samples (i.e., Plaque: non-Amish 35.62%, Amish 29.47%; Saliva: non-Amish 50.33%, Amish 35.96%). Taxa not represented by the dominant 5 phyla comprised less than 1% of the Amish and non-Amish samples (Fig 3). The only phylum unique to the Amish samples was Gemmatimonadetes.

### Core microbiome and community composition

**Fig 4:**
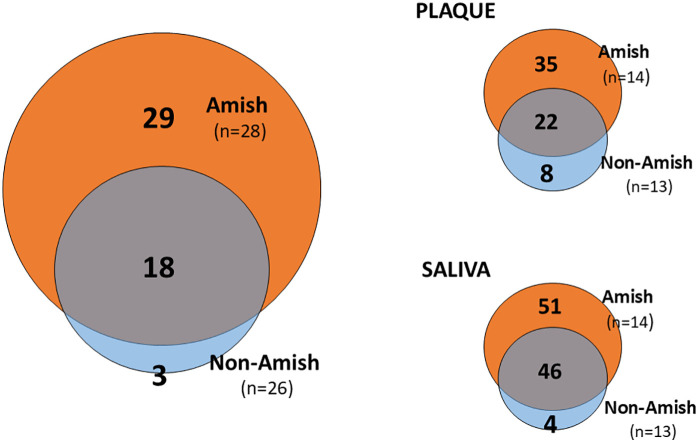
The number of contributing taxa to core microbiome of Amish and non-Amish samples, as well as separated by type (i.e., saliva or plaque). The core microbiome was determined based on presence in 100% of the samples. Orange indicates number of taxa found in the Amish samples, blue indicates number of taxa found in the non-Amish samples, and the intersection of the two shows the proportional overlap of taxa. Figure developed using BioVenn (2007-2019, Tim Hulsen).

Both Amish and non-Amish samples had core microbiomes with particular bacteria present in all the samples. The non-Amish population had a much smaller core microbiome than the Amish (21, 47 respectively, Fig 4). Only 3 of the OTUs in the core microbiome of the non-Amish were not found in the Amish samples. These three OTUs associated with *Rothia* sp., *Veillonella dispar*, and *Campylobacter* sp. Taxa present in both populations based on OTUs included *Actinomyces* sp., *Rothia dentocariosa*, *Prevotella melaninogenica*, *Streptococcus* sp., Gemellaceae, *Veillonella parvula*, *V. dispar* Type 2, *Fusobacterium* sp., *Leptotrichia* sp., *Neisseria subflava*, *Camplylobacter* sp. var 2, and *Haemophilus* sp. The pattern of overlapping core microbiomes of the Amish and non-Amish, greater taxa richness associated with the Amish bacterial oral microbiome, and subsequently smaller number of taxa unique to the non-Amish population were also observed in samples analyzed by group and type (Fig 4). In saliva samples, the core Amish microbiome was comprised of 97 OTUs, whereas the non-Amish core microbiome was comprised of only 50 OTUs; only 4 of which were unique to the non-Amish saliva core microbiome. Likewise, the core microbiome of Amish plaque samples was comprised of 57 OTUs, which included 22 of the 30 OTUs found in the core microbiome of non-Amish plaque samples. The greater richness of the Amish samples included unique OTUs associated with the phyla Actinobacteria, Bacteroidetes, Firmicutes, Fusobacteria, as well as the alpha-, beta-, and gamma- Proteobacteria.

Within the Bacteroidetes, Amish had significantly more bacteria identified as belonging to the family Weeksellaceae (p = 0.024). Leptotrichia, a genus of the Fusobacteria, was also more abundant in the Amish samples (p = 0.011). Bacteria associated with the candidate phylum GN02, class BD1–5, and β-proteobacteria of the family Neisseraceae were also significantly more abundant in Amish than non-Amish samples (p < 0.001, p = 0.011 respectively).

**Fig 5:**
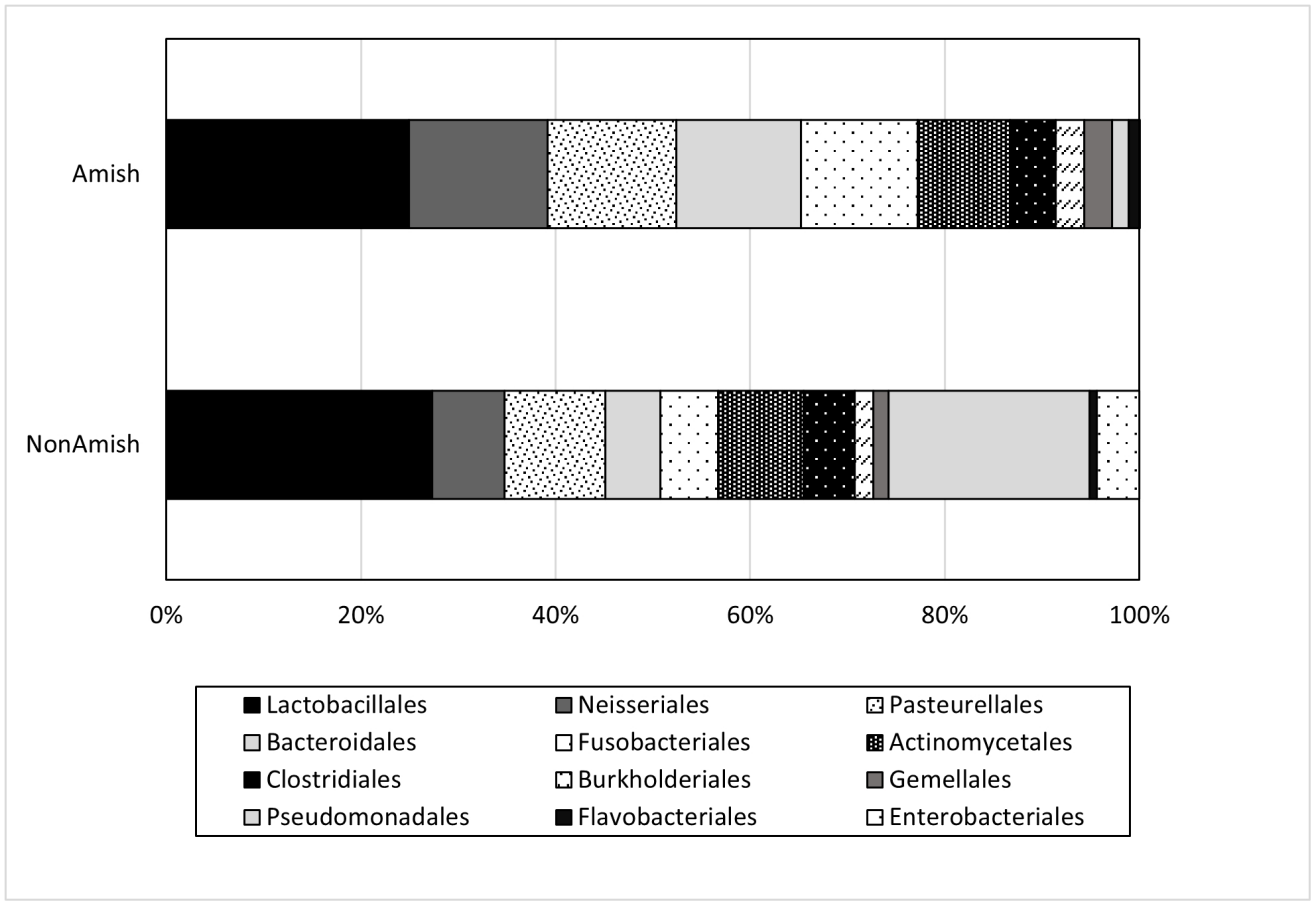
Relative abundances of 12 bacterial orders contributing 1% or more to the samples. Sequences were matched to Greengenes database using Quantitative Insights into Microbial Ecology (QIIME 1.9.1) for order-level taxonomic identification. Data are presented as stacked bars and ordered in decreasing abundance as found in the Amish sample.

**Fig 6:**
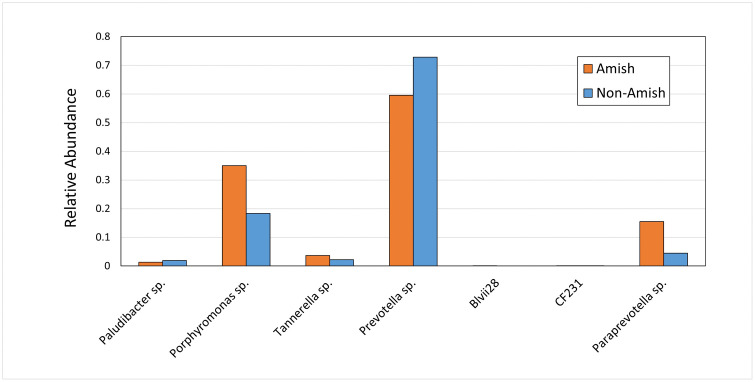
Relative abundances of the different genera from the order Bacteroidales. Sequences were matched to Greengenes database using Quantitative Insights into Microbial Ecology (QIIME 1.9.1) for genus-level taxonomic identification. Data are presented as bars with orange indicating Amish samples and blue indicating non-Amish samples.

Difference in group significance, core microbiome, phyla distribution, and community composition were also evident when analyzing the relative contributions of the Orders. While each order contributed less than 1% to the samples, Amish samples were enriched with Campylobacterales, Cardiobacteriales, Spirochaetales, Cytophagales, Rhodospirillales and Deinococcales; contrarily Bacillales and Bifidiobacteriales were more abundant in the non-Amish samples. Of the 12 orders contributing 1% or more to the samples, the relative contribution of the Bacteroidales, Gemellales, Fusobacteriales, Neisseriales, and Pasteurellales was greater in Amish samples than in the non-Amish samples (Fig 5). Although the Bacteroidales as an Order comprised a greater percentage of the taxa in Amish samples, there were observed differences at the level of genera. For example, *Porphyromonas* sp., *Paraprevotella* sp., and *Tannerella* sp. were all relatively more abundant in the Amish samples, whereas *Prevotella* sp. was more abundant in the non-Amish samples (Fig 6). Clostridiales, Enterobacteriales, and Pseudomonadales also contributed more to the relative abundance in the non-Amish samples.

## Discussion

Our study findings, even with a small sample size of 27 individuals, support differences between the oral microbiomes of the Old Order Amish and rural non-Amish residing in the same geographic area, which may be attributed to differences in genetic composition, lifestyle, and oral health hygiene [16,20]. The Old Order Amish constitute a socially isolated community with little genetic inflow, resulting in genetic distributions that differ from the non-Amish [26,27]. Lifestyle differences include limited use of modern technological advances by the Old Order Amish [28] and self-reported survey data supported significant differences in oral health habits such as frequency of dental visits and fluoride exposure, but not teeth brushing (Table 1). Saliva and plaque samples collected from the Amish had significantly greater taxonomic richness and taxonomic composition that differentiated them from the non-Amish samples (Figs 1–6).

The oral microbiome of the Amish, like their non-Amish counterparts and in agreement with eHOMD data, was dominated by the same five phyla as present in other populations [2,7,9,12]. In both the Amish and non-Amish, Firmicutes and Proteobacteria were the two major phyla represented in the samples. The Firmicutes comprised about one-third of the relative composition in both populations. The Proteobacteria, an extremely diverse phylum containing both pathogenic and non-pathogenic bacteria, however comprised a relatively smaller proportion in the Amish population compared to the non-Amish (29.5%, 35.6%, plaque and 36.0%, 50.3% saliva respectively; Fig 3). Also, the Actinobacteria comprised only a small percent in the Amish saliva samples compared to non-Amish saliva samples (Fig 3). Actinobacteria include the genera *Corynebacterium*, *Propionibacterium*, *Rothia*, *Actinomyces*, and *Bifidobacterium*, which are not often associated with gum disease [28,29]. The greater proportion of Actinobacteria in the non-Amish saliva samples as well as the relatively lower numbers of the periodontal disease associates, *Porphyromonas* spp. and *Tannerella* spp., may reflect greater dental hygiene practices of the non-Amish (Fig 6; [28,29]). While more research is needed, these differences may also be early biological indicators of the onset of periodontal disease in Amish, as differences in the microbiota of the saliva and supragingivus may influence subgingival outcomes such as periodontal disease [9,30]. Greater examination of anaerobic species within the plaque (i.e., supragingival biofilm) may further elucidate risk of periodontal disease in the two populations.

The Amish oral microbiome was significantly more taxa rich, and its taxonomic composition differed from the non-Amish samples (Figs 1 and 2). Non-Amish plaque samples showed the lowest richness, which was inversely correlated with the frequency of dental care (Table 1 and Fig 1). Given the low genetic variability of the Amish (a founder population), their social cohesiveness and homogenous lifestyle, the observation of higher taxa richness in the Amish oral bacterial microbiome is notable and may reflect factors such as exposure to farm life, as well as differences in oral hygiene [31]. A similar pattern has been reported in gut microbiomes, where individuals of the same ethnicity with the lowest economic incomes exhibited the greatest taxa richness [18]. It is possible, limited dental care and infrequent plaque removal, as observed in our Amish population, may allow substantial biofilm growth, indicating differences in the successional state of the oral biofilm. Conversely, the low richness in non-Amish samples, particularly plaque, may reflect frequent and intensive disturbance through oral health practices.

Whether the relatively greater diversity of the Amish oral microbiome influences health status is beyond the scope of this study; however, greater microbiome diversity, particularly in the gut, is generally associated with improved health. In this context, it has been previously shown that the Lancaster Amish have a relatively low prevalence of type 2 diabetes, hypertension, and high cholesterol (despite a high frequency of familial hypercholesterolemia) compared with non-Amish Europeans in the United States [32]. On the other hand, greater oral microbiome diversity could result from poorer oral health care. Amish children are less likely to receive dental care than non-Amish children [33] and generally exhibit poorer dental health [20,34]. Further research is needed to clarify the relationship between oral microbiome diversity and health outcomes in the Amish.

Overall, the Amish oral microbiome was significantly different than the non-Amish samples (ADONIS, p < 0.001). The core microbiome of the Amish was much richer than the non-Amish, housing the majority of unique taxa. For example, only Amish samples included OTU from the Gemmatimonadetes, which is a phylum of bacteria typically found in soil or associated with plant roots [35,36]. Gemmatimonadetes, which are largely chemoorganoheterotrophs, have been in some cases associated with periodontal disease [7]. The Amish samples also included more Bacteroidales, Gemellales, Fusobacteriales, Pastuerellales, and Neisseriales, whereas the non-Amish individual samples were enriched in Pseudomonadales and Enterobacteriales (Fig 5). While these differences in samples clearly delineate the two populations, understanding what these differences mean to overall health, if any, still needs to be elucidated.

Even with high quality sequence data, mapping only the v4 section of the 16S rRNA gene and using OTU rather than ASV-based methods does not permit us to separate closely related species. Therefore, species such as *Streptococcus mutans* and *S. gordonii*, which play very different roles in oral health, could not be fully distinguished. Other study limitations, all of which could contribute to sources of uncontrolled variability, included small sample size, the possibility of selection bias by recruiting individuals from two different settings (i.e., routine dental care and Maryland Amish Research Clinic), and limited information collected from health surveys. Furthermore, our understanding of the functional roles of the more than 700 phylotypes registered in the eHOMD is extremely limited. Here we have taken the first step, characterizing the oral microbiome of the Old Order Amish and comparing it to others that live in close geographic proximity but differ in genetics, lifestyle habits, and dental hygiene. Further work is now needed to better understand these differences and their role in oral health.

## Conclusion

As a preliminary study, we can conclude that there are significant differences of the oral microbiome between the rural non-Amish and the Old Order Amish, an ethnoreligious founder population with a relatively homogeneous lifestyle and who are socially cohesive. Although several molecular studies have been conducted on the gut microbiota in Old Order Amish, few to no molecular analyses have been conducted on the microbiota of the oral cavity. This study provides an initial molecular characterization of the Amish oral microbiome and compares it to local, rural non-Amish. Further study and data analyses are needed to elucidate the role genetics, lifestyle, and oral health habits contribute to shaping the microbiome and health outcomes. Analyses of the phylogenetic relatedness of the different biomes and functional analysis may also help elucidate our understanding of the microbiomes in relationship to periodontal disease.

This initial characterization of the Amish oral microbiome demonstrates that there is a core oral microbiome, and that the oral microbiome of the Amish and non-Amish are significantly different. Furthermore, we provide evidence that the Amish microbiome includes many of the microorganisms found in the oral cavity of the non-Amish, but with notably greater diversity. These differences reflect differences in lifestyle, oral health habits, and genetic composition.
